# Association of Smoking, Alcohol, and Obesity with Cardiovascular Death and Ischemic Stroke in Atrial Fibrillation: The Atherosclerosis Risk in Communities (ARIC) Study and Cardiovascular Health Study (CHS)

**DOI:** 10.1371/journal.pone.0147065

**Published:** 2016-01-12

**Authors:** Younghoon Kwon, Faye L. Norby, Paul N. Jensen, Sunil K. Agarwal, Elsayed Z. Soliman, Gregory Y. H. Lip, W. T. Longstreth, Alvaro Alonso, Susan R. Heckbert, Lin Y. Chen

**Affiliations:** 1 Division of Cardiovascular Medicine, Department of Medicine, University of Minnesota, Minneapolis, Minnesota, United States of America; 2 Division of Epidemiology and Community Health, School of Public Health, University of Minnesota, Minneapolis, Minnesota, United States of America; 3 Department of Epidemiology, University of Washington, Seattle, Washington, United States of America; 4 Department of Medicine, Johns Hopkins University, Baltimore, Maryland, United States of America; 5 Epidemiological Cardiology Research Center (EPICARE), Department of Epidemiology and Prevention, and Department of Medicine-Cardiology, Wake Forest School of Medicine, Winston-Salem, North Carolina, United States of America; 6 University of Birmingham Centre for Cardiovascular Sciences, City Hospital, Birmingham, United Kingdom; 7 Aalborg Thrombosis Research Unit, Department of Clinical Medicine, Aalborg University, Aalborg, Denmark; 8 Department of Neurology, University of Washington, Seattle, Washington, United States of America; Osaka University Graduate School of Medicine, JAPAN

## Abstract

Atrial fibrillation (AF) is associated with an increased risk of ischemic stroke and cardiovascular (CV) death. Whether modifiable lifestyle risk factors are associated with these CV outcomes in AF is unknown. Among Atherosclerosis Risk in Communities (ARIC) study and Cardiovascular Health Study (CHS) participants with incident AF, we estimated the risk of composite endpoint of ischemic stroke or CV death associated with candidate modifiable risk factor (smoking, heavy alcohol consumption, or high body mass index [BMI]), and computed the C-statistic, net reclassification improvement (NRI), and integrated discrimination improvement (IDI) of incorporating each factor into the CHA_2_DS_2_-VASc. Among 1222 ARIC (mean age: 63.4) and 756 CHS (mean age: 79.1) participants with incident AF, during mean follow-up of 6.9 years and 5.7 years, there were 332 and 335 composite events respectively. Compared with never smokers, current smokers had a higher incidence of the composite endpoint in ARIC [HR: 1.65 (1.21–2.26)] but not in CHS [HR: 1.05 (0.69–1.61)]. In ARIC, the addition of current smoking did not improve risk prediction over and above the CHA_2_DS_2_-VASc. No significant associations were observed with alcohol consumption or BMI with CVD outcomes in AF patients from either cohort. Smoking is associated with an increased risk of ischemic stroke or CV death in ARIC, which comprised mostly middle-aged to young-old (65–74 years), but not in CHS, which comprised mostly middle-old or oldest-old (≥75 years) adults with AF. However, addition of smoking to the CHA_2_DS_2_-VASc score did not improve risk prediction of these outcomes.

## Introduction

Atrial fibrillation (AF) affects 2.3 million in the US and is associated with 4–5 fold increased risk of ischemic stroke and two-fold risk of death.[1, 2] Due to serious long-term disability associated with ischemic stroke, risk prediction tools such as CHADS_2_ (congestive heart failure, hypertension, age ≥75 years, diabetes mellitus, previous stroke/transient ischemic attack [TIA]) and CHA_2_DS_2_-VASc (congestive heart failure, hypertension, age ≥75 years, diabetes mellitus, previous stroke/TIA, vascular disease, age 65–74 years, female sex) are increasingly used in clinical practice to assess the risk of stroke and thromboembolism among patients with AF.[3–5] These risk scores have also been shown to predict death in patients with AF.[6, 7] Most of the variables considered in these schemes, however, are not modifiable risk factors. Identification of modifiable risk factors may potentially inform novel prevention strategies. Smoking, alcohol consumption, and weight represent potentially modifiable lifestyle risk factors and have been investigated extensively in relation to cardiovascular (CV) disease in the general population. However, data on their prognostic implication in patients with AF are relatively limited.

In this study, we aimed to determine whether these modifiable risk factors are associated with increased risk of incident ischemic stroke or CV death and whether addition of these factors improves risk prediction over and above CHA_2_DS_2_-VASc in participants with incident AF in two large community-based cohorts, the Atherosclerosis Risk in Communities (ARIC) Study and the Cardiovascular Health Study (CHS).

## Methods

**Fig 1:**
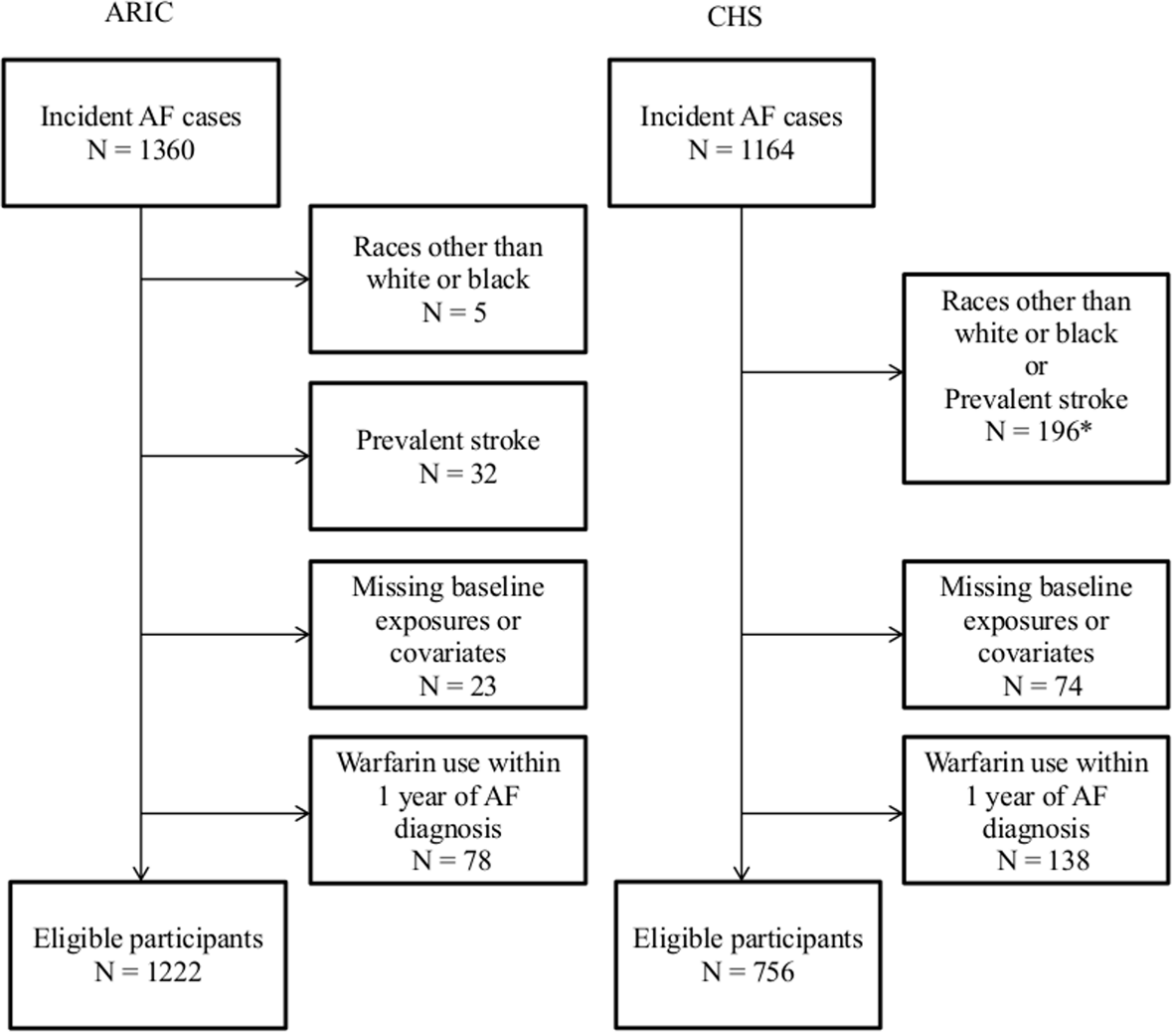
Flow chart depicting inclusion and exclusion of the participants in each cohort. *Medicare-enhanced data are not presented separately for exclusions due to races other than white or black and exclusions due to prevalent stroke, due to a small sample. ARIC, Atherosclerosis Risk in Communities; CHS, Cardiovascular Health Study.

The designs of the ARIC study and CHS have been previously described.[8, 9] Both studies share similar objectives to identify risk factors of atherosclerosis and CV disease. In ARIC, at baseline, 15,792 middle-aged participants (45–64 years old) were recruited from four US communities (Forsyth County, NC; Jackson, MS; Minneapolis suburbs, MN; and Washington County, MD) between 1987 and 1989. After the baseline examination, participants had 4 additional exams, the last occurring in 2011−2013. In addition to study exams, ARIC participants have received annual follow-up calls since the first visit to collect information on general health and hospitalizations. For this study, we included participants with incident AF between baseline (1987–89) and the end of 2006 to allow at least 5 years of follow up after AF diagnosis. CHS is a study of risk factors for coronary heart disease (CHD) and stroke in older people that enrolled 5201 participants aged ≥65 years from Medicare eligibility lists between 1989–1990 at 4 field centers (Forsyth County, NC; Sacramento County, CA; Washington County, MD; and Pittsburgh, PA). To enhance minority representation, during 1992–1993, 687 black participants were recruited. The baseline visit and annual study visits through 1998–99 included interviews, laboratory measurements, electrocardiograms (ECG), and clinic examinations. Study visits alternated every six months with telephone calls until 1998–99; thereafter, participants were contacted by phone every 6 months. For this study, we included participants with incident AF from baseline (1989–90 or 1992–1993) through the end of 2000. Participants with prevalent AF or atrial flutter at baseline, stroke that occurred prior to incident AF, body mass index less than 18.5 (kg/m^2^) or race/ethnicity other than white or black were excluded. Those with missing data on covariates, smoking status, alcohol consumption, or weight and height, and with any documented evidence of warfarin use within 1 year of AF ascertainment were also excluded. After exclusions, 1222 ARIC and 756 CHS participants were eligible for the analysis (Fig 1). Written informed consent was obtained from all participants and the study was approved by institutional review boards by each participating study field center and coordinating center [ARIC study field centers (the Universities of NC, MS, MN, and John Hopkins University); ARIC study coordinating center (University of NC); CHS field centers (University of CA-Davis, University of Pittsburgh, Johns Hopkins University, and Wake Forest University); CHS coordinating center (University of Washington)]. The research was conducted in accordance with the principles described in the Declaration of Helsinki.

In ARIC, incident AF diagnoses were obtained from two sources: 12-lead resting ECGs at follow up study exams and hospital discharge records (*International Classification of Disease* (ICD)*-*9 code 427.31 and 427.32 –AF/atrial flutter).[10] Annual follow-up telephone calls to participants and survey of local hospitals were used to identify hospitalizations or deaths. The sensitivity and specificity of hospital discharge diagnoses for AF was found to be 84% and 98%, respectively, in a previous ARIC study.[10]AF ascertainment in CHS was similar to the ARIC study. Resting 12-lead ECGs were performed at annual visits through 1999. At each follow up contact, participants were asked to report all hospitalizations. Hospital discharge records were obtained and were supplemented with Medicare inpatient and outpatient claims data. AF was defined by a study ECG, a hospital discharge diagnosis, or a Medicare inpatient or outpatient claim with ICD-9 codes for AF or atrial flutter. AF that occurred during the same hospital stay as coronary artery bypass surgery or valve surgery was not included. The positive predictive value of hospital discharge diagnosis was 98.6% for diagnosis of AF in a prior CHS study.[11]

Smoking status was categorized as never, former or current based on self-report. Alcohol consumption was ascertained by means of an interviewer-administered dietary questionnaire. Based on a previous ARIC report, participants were classified into three alcohol-use groups: no use; light to moderate use, 1–98 g per week for women and 1–196 g per week for men; heavy use, more than 98 gram per week for women and 196 g per week for men.[12] In calculating the amount of alcohol consumed, we assumed that 4 oz of wine contains 10.8 g, 12 oz of beer contains 13.2 g, and 1.5 oz of liquor contains 15.1 g of ethanol. Body mass index (BMI: kg/m^2^) was calculated as weight (in kilograms) divided by height (in meters) squared and was divided into three categories: normal, 18.5< BMI<25; overweight, 25≤ BMI<29.9; obese, BMI ≥ 30. Information on each of the exposure variables was obtained from the closest study visit prior to AF ascertainment. The mean interval between acquisition of exposure variable information and AF diagnosis was 4.5 years in ARIC and 0.6 years in CHS.

Information on CHA_2_DS_2_-VASc variables was obtained from the closest study visit prior to AF ascertainment. In both ARIC and CHS, hypertension was defined as systolic blood pressure (BP) ≥140 mmHg and/or diastolic BP ≥90 mmHg, or BP medication use in the past 2 weeks.

In ARIC, diabetes was present if one of the following criteria was met; 1) fasting glucose ≥ 126 mg/dL or non-fasting glucose ≥ 200 mg/dL, 2) self-reported physician diagnosis of diabetes, or 3) currently taking medication for diabetes. In CHS, diabetes was present if fasting serum glucose level ≥126 mg/dL or the participant was currently taking medication for diabetes. In ARIC, prevalent CHD included a history of myocardial infarction (MI), MI indicated on the baseline ECG, or history of revascularization. Prevalent heart failure (HF) was identified by the Gothenburg criteria[13] or self-report of HF medication use in the past 2 weeks. During follow-up, prevalent HF at each visit was identified as having a hospitalization with an ICD 9–428.0 code during follow-up prior to that exam. In CHS, prevalent CHD included a positive history for MI, coronary revascularization, or angina using information from self-report and hospitalization records. HF was identified by physician diagnosis and one of the following: 1) documented symptoms (e.g., shortness of breath, fatigue, orthopnea, paroxysmal nocturnal dyspnea) or signs (e.g., edema, pulmonary rales, gallop rhythm) consistent with HF; 2) supporting clinical findings such as pulmonary edema on chest x-ray; or 3) therapy for HF, including diuretics, digitalis, angiotensin-converting enzyme inhibitors, or beta-blockers. Peripheral artery disease (PAD) was defined as an ankle-brachial index (ABI) <0.9 assessed at visit 1, 3 or 4 (ARIC) or at the baseline examination (CHS). PAD was also defined by the presence of any of the following: PAD-related hospitalization, amputation, leg revascularization, or intermittent claudication as assessed by annual questionnaire.[14]

In ARIC, incident ischemic stroke and CV death were identified through December 31, 2011 annual telephone interviews, study visits, surveillance of the ARIC community hospitals for all participants’ hospitalizations.[15] Hospital reports were reviewed if the discharge diagnosis included a cerebrovascular disease (ICD-9 codes 430 to 438 or ICD-10 I60-I69), if a cerebrovascular procedure was mentioned in the summary, or if the computerized tomography or magnetic resonance image report showed evidence of cerebrovascular disease. ARIC adapted the National Survey of Stroke criteria for its stroke definition. Both definite and probable ischemic stroke were included. Only incident ischemic stroke events that occurred after ascertainment of AF were included for our analyses. Hemorrhagic stroke events were censored. In ARIC, information on death was obtained from the National Death Index and state death registries.CV death was defined as death with ICD‐9 code 401‐459 or ICD‐10 code I10‐I99. A computerized algorithm and physician reviewer independently confirmed the diagnosis of ischemic stroke and CV death.[16]

In CHS, incident ischemic stroke and CV death from January 1, 1989 through December 31, 2011 were identified at 6-monthly contacts.[17] Stroke cases were then adjudicated by physicians based on the information from patient interviews, medical records, and brain-imaging studies if available. Ischemic stroke was differentiated from hemorrhagic stroke as previously reported.[18] CV death was ascertained in similar manner to ARIC and included atherosclerotic coronary disease, cerebrovascular disease (stroke), other atherosclerotic disease (such as aortic aneurysm), and other vascular disease (such as valvular heart disease or pulmonary embolism).[19]

The primary outcome was a composite outcome of ischemic stroke and CV death. Secondary outcomes were individual components of the primary outcome (i.e. ischemic stroke and CV death). Incidence rates of outcomes were expressed as number of events per 1,000 person-years. Person-years at risk were calculated from AF ascertainment until date of development of stroke, death, loss to follow up, or end of follow up, whichever occurred first. We estimated the association of smoking and drinking habits, and BMI with incident ischemic stroke or CV death using Cox proportional hazard models adjusted for age, sex and race, and the subsequent model adjusted for race and CHA_2_DS_2_-VASc variables excluding history of stroke or TIA (age, sex, HF, hypertension, diabetes, previous MI and PAD; Hereafter called ‘CHA_2_DS_2_-VASc adjusted model’). Survival curves were calculated from stratified Cox regression models, and were adjusted for age, sex and race. Interaction with race and sex was tested by including cross-product terms. For predictors with a significant association, we also tested whether addition of each variable improved risk prediction of 5-year IS risk above and beyond CHA_2_DS_2_-VASc. To assess model discrimination, we computed the C-statistic using methods that accounted for censoring. To test model calibration, “goodness-of-fit” of the observed and expected number of events within estimated risk decile groups was compared using the Grønnesby-Borgan statistic.[20] Finally, to assess improvement in risk classification, net reclassification improvement (NRI)[21] and relative integrated discrimination improvement (IDI)[22] for 5-year risk prediction were calculated.

## Results

The total number of participants included in this analysis were 1222 for ARIC and 756 for CHS.

Characteristics of participants close to the time of AF diagnosis are presented in Table 1. Participants in the ARIC study were younger and had a lower prevalence of CV comorbidities. In ARIC, during 8504 person-years of follow-up (median 5.6 years), there were 332 events (composite of ischemic stroke and CV death). In CHS, during 4230 person-years of follow-up (median 4.4 years), there were 335 events.

**Table 1:** Characteristics of participants at the visit prior to atrial fibrillation diagnosis, Atherosclerosis Risk in Communities (ARIC) study from 1987 to 2006 and Cardiovascular Health Study (CHS) from 1989 to 2006.

		ARIC	CHS
N		1222	756
Age, years, mean (SD)		63.4 (6.2)	79.1 (6.2)
Women		43.0%	49.6%
Race	Black	18.9%	2.8%
Smoking	Current	23.3%	8.7%
Smoking	Past	44.5%	47.1%
Smoking	Never	32.2%	44. 2%
Alcohol	No	66.1%	71.3%
Alcohol	Light to moderate	26.9%	+
Alcohol	Heavy	7%	+
BMI	Mean (SD)	29.7 (6.1)	26.1 (4.6)
BMI	Median	28.5	25.7
Diabetes		25.8%	27.0%
Hypertension		59.1%	74.1%
Myocardial infarction		13.3%	18.0%
Heart failure		12.4%	16.9%
Peripheral artery disease		5.9%	26.5%
Aspirin use		59.3%	48.9%

^+^Medicare-enhanced data are not presented separately for light to moderate and heavy alcohol use due to a small sample size. Combined proportion is 28.6%. Data are presented as % of participants unless otherwise stated.

Table 2 shows the crude incidence rates and hazard ratios (HR) for the composite endpoint of ischemic stroke and CV death based on the three exposure variables.

**Table 2:** Association of Smoking, Alcohol and Body Mass Index with Composite Endpoint of Ischemic Stroke and Cardiovascular Death in Participants with Incident Atrial Fibrillation, Atherosclerosis Risk in Communities (ARIC) study from 1987 to 2006 and Cardiovascular Health Study (CHS) from 1989 to 2006.

	ARIC: HR (95% CI)			CHS: HR 95% CI)		
	Smoking					
	Never	Past	Current	Never	Past	Current
Cardiovascular deaths or ischemic stroke (N)	100	150	82	139	149	25
Person years	2959.8	3980.3	1564.2	2013.3	1855.1	309.8
Incidence rates (95% CI)*	33.8 (27.6–40.9)	37.7 (32.0–44.1)	52.4 (42.0–64.7)	69.0 (58.5–81.5)	80.3 (68.4–94.3)	80.7 (54.5–119.4)
HR (95% CI) Model 1	1	1.13 (0.87–1.48)	1.57 (1.16–2.12) †	1	1.14 (0.89–1.47)	1.27 (0.83–1.92)
HR (95% CI) Model 2	1	1.09 (0.83–1.43)	1.65 (1.21–2.26) †	1	1.04 (0.81–1.34)	1.05 (0.69–1.61)
	Alcohol					
	No	Light to moderate	Heavy	No	Light to moderate	Heavy
Cardiovascular deaths or ischemic stroke (N)	217	88	27	241	+	+
Person years	5341.7	2561.9	600.8	2968.4	+	+
Incidence rates (95% CI)*	40.6 (35.5–46.3)	34.3 (27.7–42.1)	44.9 (30.3–64.4)	81.2 (71.6–92.1)	71.6 (58.4–87.7)	30.2 (4.3–214.2)
HR (95% CI) Model 1	1	0.93 (0.72–1.21)	1.19 (0.79–1.79)	1	0.84 (0.66–1.06)	0.52 (0.06–4.93)
HR (95% CI) Model 2	1	1.04 (0.79–1.35)	1.19 (0.75–1.88)	1	1.01 (0.79–1.29)	0.61 (0.07–5.24)
	BMI					
	18.5 to <25	25 to < 30	> = 30	18.5 to <25	25 to < 30	> = 30
Cardiovascular deaths or ischemic stroke (N)	57	122	153	125	136	63
Person years	1874.9	3366.7	3262.7	1549.3	1884.6	799.9
Incidence rates (95% CI)*	30.4 (23.3–39.1)	36.2 (30.2–43.1)	46.9 (39.9–54.8)	80.7 (67.7–96.1)	72.2 (61.0–85.4)	78.8 (61.5–100.8)
HR (95% CI) Model 1	1	1.14 (0.84–1.56)	1.42 (1.05–1.93)	1	0.92 (0.72–1.17)	1.12 (0.82–1.54)
HR (95% CI) Model 2	1	0.97 (0.71–1.33)	0.96 (0.69–1.32)	1	0.83 (0.64–1.06)	1.02 (0.74–1.41)

*per 1,000 person-years. Model 1 adjusted for age, race and sex. Model 2 adjusted for Model 1 + CHA_2_DS_2_ -VASc variables (congestive heart failure, hypertension, age ≥75 years, diabetes mellitus, vascular disease, age 65–74 years, sex class (female)) all as separate variables, not a score) excluding prior Transient ischemic attack/ stroke. Median follow up was 6.6 years for ARIC and 4.4 years for CHS.

† Indicates P < .05. ARIC, Atherosclerosis Risk in Communities; CHS, Cardiovascular Health Study. ^+^Medicare-enhanced data are not presented in these cells due to a small sample size.

**Fig 2:**
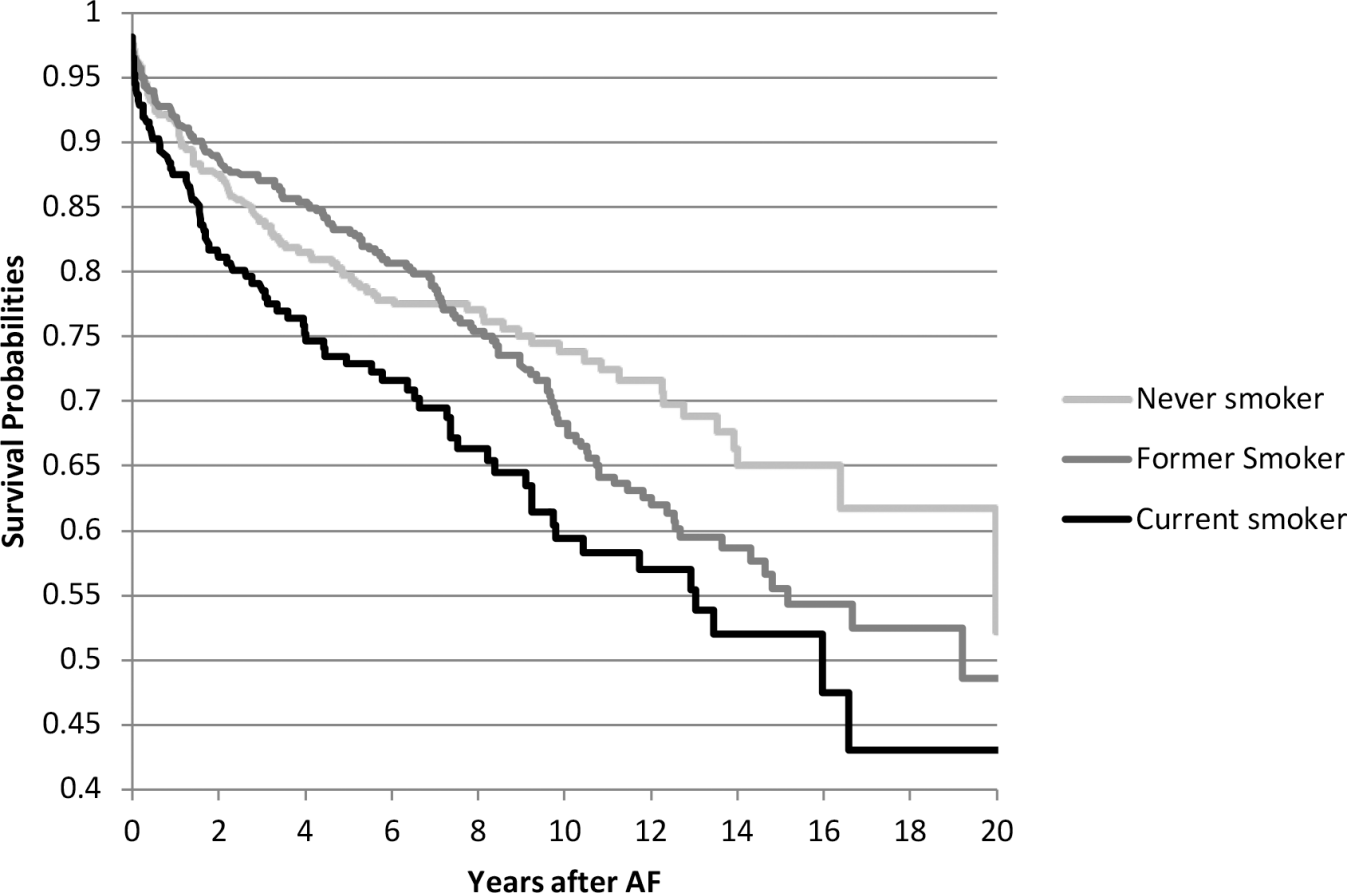
Survival free probability of the composite endpoint of ischemic stroke and cardiovascular death in participants with incident atrial fibrillation, by smoking status (Atherosclerosis Risk in Communities [ARIC] Study).

In ARIC, current smoking (vs. never smoking) was associated with higher risk of the composite endpoint in both the demographic adjusted and CHA_2_DS_2_-VASc adjusted models (HR 1.57; 95% CI, 1.16–2.12 and HR 1.65; 95% CI, 1.21–2.26, respectively) (Fig 2). Obesity (vs. normal weight) was associated with a higher risk of ischemic stroke and CV death but this association was no longer significant after further adjustment for CHA_2_DS_2_-VASc variables. No significant association was found with alcohol intake. In CHS, none of the modifiable risk factors was associated with the composite outcome. Associations did not differ by sex or race. In ARIC, the association between smoking and the composite outcome was mainly driven by CV death, which accounted for 270 events (81%) out of total 332 composite outcome events) (Tables 3 and 4). In CHS, smoking was significantly associated with CV death but not with ischemic stroke. Alcohol consumption and BMI were not associated with the individual components of the composite outcome: ischemic stroke or CV death (Tables 3 and 4).

**Table 3:** Association of smoking, alcohol and body mass index with ischemic stroke in participants with incident atrial fibrillation, Atherosclerosis Risk in Communities (ARIC) study from 1987 to 2006 and Cardiovascular Health Study (CHS) from 1989 to 2006.

	ARIC: HR (95% CI)			CHS: HR (95% CI)		
	Smoking					
	Never	Past	Current	Never	Past	Current
Ischemic stroke (N)	36	44	19	57	+	+
Person years	3164.1	4433.1	1863.8	2086.3	+	+
Incidence rates (95% CI)*	11.38 (8.10–15.57)	9.93 (7.30–13.20)	10.19 (6.34–15.59)	27.32 (21.07–35.42)	21.56 (15.88–29.28)	25.59 (12.8–51.18)
HR (95% CI) Model 1	1	0.94 (0.59–1.50)	0.91 (0.52–1.62)	1	0.84 (0.55–1.28)	0.97 (0.45–2.08)
HR (95% CI) Model 2	1	0.89 (0.56–1.44)	0.92 (0.51–1.65)	1	0.76 (0.49–1.17)	0.81 (0.37–1.81)
	Alcohol					
	No	Light to moderate	Heavy	No	Light to moderate	Heavy
Ischemic stroke (N)	65	25	9	74	32	0
Person years	6016.6	2743.2	701.1	2964.2	1299.5	33.1
Incidence rates (95% CI)*	10.80 (8.41–13.68)	9.11 (6.04–13.24)	12.83 (6.34–23.43)	24.96 (19.88–31.35)	24.63 (17.41–34.82)	N/A
HR (95% CI) Model 1	1	0.95 (0.58–1.55)	1.29 (0.64–2.63)	1	1.06 (0.69–1.63)	N/A
HR (95% CI) Model 2	1	1.05 (0.64–1.72)	1.32 (0.65–2.70)	1	1.12 (0.73–1.73)	N/A
	BMI					
	18.5 to <25	25 to < 30	> = 30	18.5 to <25	25 to < 30	> = 30
Ischemic stroke (N)	18	36	45	39	44	18
Person years	2114.8	3688.9	3657.2	1545.1	1884.6	799.9
Incidence rates (95% CI)*	8.13 (5.00–12.59)	9.76 (6.95–13.35)	12.31 (9.09–16.31)	25.24 (18.44–34.55)	23.35 (17.37–31.37)	22.5 (14.18–35.72)
HR (95% CI) Model 1	1	1.17 (0.66–2.06)	1.37 (0.79–2.39)	1	0.97 (0.63–1.52)	0.97 (0.55–1.71)
HR (95% CI) Model 2	1	0.99 (0.56–1.77)	0.98 (0.55–1.76)	1	0.87 (0.55–1.37)	0.92 (0.51–1.63)

*per 1,000 person-years.

Model 1 adjusted for age, race and sex. Model 2 adjusted for Model 1 + CHA_2_DS_2_-VASc variables (congestive heart failure, hypertension, age ≥75 years, diabetes mellitus, vascular disease, age 65–74 years, sex class (female)) all as separate variables, not a score) excluding prior Transient ischemic attack/ stroke. Median follow up was 6.6 years for ARIC and 4.4 years for CHS. ARIC, Atherosclerosis Risk in Communities; CHS, Cardiovascular Health Study; N/A, not applicable. ^+^Medicare-enhanced data are not presented in these cells due to a small sample size.

**Table 4:** Association of smoking, alcohol and body mass index with cardiovascular death in participants with incident atrial fibrillation, Atherosclerosis Risk in Communities (ARIC) study from 1987 to 2006 and Cardiovascular Health Study (CHS) from 1989 to 2006.

	ARIC: HR (95% CI)			CHS: HR (95% CI)		
	Smoking					
	Never	Past	Current	Never	Past	Current
Cardiovascular deaths (N)	77	121	72	129	141	25
Person years	3102.3	4151.9	1615.9	2235.7	2025.4	328.6
Incidence rates (95% CI)*	24.8 (19.7–30.8)	29.1 (24.3–34.7)	44.6 (35.1–55.8)	57.7 (48.6–68.6)	69.6 (59.0–82.1)	76.1 (51.4–112.6)
HR (95% CI) Model 1	1	1.18 (0.88–1.60)	1.87 (1.34–2.60)†	1	1.23 (0.95–1.59)	1.55 (1.00–2.38)†
HR (95% CI) Model 2	1	1.15 (0.85–1.57)	2.06 (1.46–2.90)†	1	1.29 (0.99–1.68)	1.64 (1.08–2.48)†
	Alcohol					
	No	Light to moderate	Heavy	No	Light to moderate	Heavy
Cardiovascular deaths (N)	176	72	22	213	+	+
Person years	5553.3	2693.6	623.2	3181.8	+	+
Incidence rates (95% CI)*	31.7 (27.3–36.6)	26.7 (21.1–33.4)	35.3 (22.8–52.5)	66.9 (58.5–76.6)	59.1 (47.5–73.5)	30.2 (4.3–214.2)
HR (95% CI) Model 1	1	0.93 (0.70–1.25)	1.20 (0.77–1.89)	1	0.83 (0.64–1.07)	0.66 (0.07–6.38)
HR (95% CI) Model 2	1	1.07 (0.80–1.44)	1.19 (0.75–1.88)	1	0.99 (0.76–1.3)	0.64 (0.07–5.75)
	BMI					
	18.5 to <25	25 to < 30	> = 30	18.5 to <25	25 to < 30	> = 30
Cardiovascular deaths (N)	44	102	124	109	117	59
Person years	1941.2	3484.3	3444.6	1661.9	2014.0	832.8
Incidence rates (95% CI)*	22.7 (16.7–30.1)	29.3 (24.0–35.4)	36.0 (30.1–42.8)	65.6 (54.4–79.1)	58.1 (48.5–69.6)	70.84 (54.9–91.4)
HR (95% CI) Model 1	1	1.23 (0.87–1.75)	1.48 (1.05–2.09)	1	0.92 (0.71–1.2)	1.27 (0.92–1.77)
HR (95% CI) Model 2	1	1.04 (0.73–1.47)	0.97 (0.67–1.39)	1	0.88 (0.67–1.14)	1.17 (0.83–1.64)

*per 1,000 person-years.

Model 1 adjusted for age, race and sex. Model 2 adjusted for Model 1 + CHA_2_DS_2_-VASc variables (congestive heart failure, hypertension, age ≥75 years, diabetes mellitus, vascular disease, age 65–74 years, sex class (female)) all as separate variables, not a score) excluding prior Transient ischemic attack/ stroke. Median follow up was 6.6 years for ARIC and 4.4 years for CHS.

† Indicates P < .05. ARIC, Atherosclerosis Risk in Communities; CHS, Cardiovascular Health Study. ^+^Medicare-enhanced data are not presented in these cells due to a small sample size.

Since smoking was the only risk factor significantly associated with the composite outcome in ARIC, we computed the C-statistic, NRI, and IDI for the addition of smoking to the CHA_2_DS_2_-VASc score in the ARIC study. Table 5 shows that with addition of smoking, there was increase in the C-statistic from 0.701 to 0.712 but both NRI and IDI were not significant

**Table 5:** Addition of smoking to CHA_2_DS_2_-VASc in prediction of 5-year risk of ischemic stroke or cardiovascular death, Atherosclerosis Risk in Communities (ARIC) study from 1987 to 2006.

	C-statistic (95% CI)	Category-based NRI (95% CI)	Relative IDI (95% CI)
CHA_2_DS_2_ -VASc	0.701 (0.665–0.737)	N/A	N/A
CHA_2_DS_2_ -VASc + Smoking	0.712 (0.677–0.746)	0.040 (-0.017 to 0.096)	0.043 (-0.012 to 0.104)

IDI, integrated discrimination improvement; NA, not applicable; NRI, net risk improvement

## Discussion

In this analysis of two large prospective, community-based studies we sought to determine whether common modifiable risk factors, smoking, alcohol consumption, and obesity, were associated with ischemic stroke or CV death in older individuals with AF. We found that in ARIC (which comprised mostly middle-aged to young-old [65–74 years] adults), but not in CHS (which comprised mostly middle-old or oldest-old [≥75 years] adults with AF), current smoking (vs. never smoking) was associated with a significantly higher risk of ischemic stroke or CV death in people with AF, and that the association in ARIC persisted after adjusting for components of the CHA_2_DS_2_-VASc. Adding smoking status to the CHA_2_DS_2_-VASc risk score, however, did not improve risk prediction of ischemic stroke or CV death. In both ARIC and CHS, alcohol consumption and BMI were not associated with ischemic stroke or CV death.

Although smoking is a well-established risk factor for both ischemic stroke and CV death in the general population, little is known about the contribution of smoking to ischemic stroke and CV death in persons with AF. In the Danish Diet Cancer and Health study, smoking was associated with a higher risk of thromboembolism or death in patients with AF after adjusting for well-recognized risk factors and the associations were strongest among women.[23] In the current study, among ARIC participants with AF, current smoking (vs. never smoking) was associated with about a 50% higher risk of composite outcome of ischemic stroke or CV death after adjusting for components of the CHA_2_DS_2_-VASc variables. The reason this association was not also observed in the CHS is unclear. As most smokers generally begin smoking at an early age, one would anticipate a greater dose exposure among older current smokers simply because of the presumed longer exposure to smoking. However a recent meta-analysis of pooled data from 12 prospective studies showed that the risk of current smoking status on the incidence of coronary heart disease tended to be attenuated with increasing age.[24] Thus, the discrepancy in our study may be related to the older age distribution in CHS: participants in the ARIC study were mostly in the middle aged to young-old category (65–74 years) as compared with CHS participants who were mostly in the middle-old (75–84 years) or oldest-old (≥85 years). Additional causes of discrepant results between the two cohorts may have been related to 1) residual ascertainment bias despite the comparable definition of exposures and outcomes, and similar observation period and 2) imbalanced proportion of warfarin users who may not have been effectively excluded by our methods. When smoking status was added to the CHA_2_DS_2_-VASc model in ARIC, however, the risk prediction was not improved as evidenced by absence of significant NRI or IDI.

Unlike smoking, no significant association was found between alcohol intake and the composite outcome of incident ischemic stroke or CV death in ARIC in those with AF. While light to moderate alcohol consumption has been associated with lower risk of CV outcomes, heavy alcohol consumption has been linked to higher risk of adverse health outcomes, including stroke and death.[25] A few studies have examined this among people with AF. In analysis of SPAF (Stroke Prevention in Atrial Fibrillation) I-III trials, a lower incidence of ischemic stroke was observed among participants who reported regular alcohol use than those who did not consume any alcohol.[26] Similarly, in SPORTIF (Stroke Prevention Using Oral Thrombin Inhibitor In Atrial Fibrillation) III and IV trials, the incidence of stroke or systemic embolism was lower in alcohol users than in non-users.[27] By contrast, heavy alcohol consumption is harmful; Overvad et al. reported a higher incidence of thromboembolic event or death among heavy alcohol users in participants with incident AF in the Danish Diet, Cancer and Health study.[28] Of note, Overvad et al. defined heavy alcohol intake as consuming more than 27 drinks (324 g equivalent) per week for men and 20 drinks (240 g equivalent) per week for women.[28] In our study, consumption of more than 196 g and 98 g alcohol per week defined heavy use of alcohol for men and women, respectively. Therefore, many of the participants likely classified as heavy alcohol users in our study may have been classified as moderate users in the study by Overvad et al., possibly explaining the discrepant results between the two studies. Furthermore even with the broader heavy drinker category in this study, the proportion of heavy drinkers was low (7%). Therefore use of a stricter definition of heavy drinkers may have resulted in too few participants in this category to yield reasonable estimates. Finally, a slightly different definition of the composite outcome (i.e., ischemic stroke and CV death in this study vs. thromboembolic event and total death by Overvad et al.) may have also played a role in yielding different results.

In our study, BMI was not significantly associated with the composite outcome in participants with AF. Increased risk of the composite outcome in the obese group in ARIC did not persist after full adjustment for CHA_2_DS_2_-VASc covariates. While obesity is predictive of a higher incidence of CV outcomes, including AF and ischemic stroke, in the general population,[29] whether it predicts poor CV outcomes in patients with established AF has not been clear. Post hoc analyses of the Atrial Fibrillation Follow-Up Investigations of Rhythm Management (AFFIRM) trial suggested an ‘obesity paradox’ with a lower risk of total or CV death in overweight and obese AF patients.[30] In contrast, the Danish Diet, Cancer and Health study reported a higher risk of ischemic stroke and thromboembolism among obese men and normal weight women with incident AF.[31] Conversely, our study suggests neither an advantage nor a disadvantage by being obese, overweight or normal weight with respect to adverse CV outcomes in those with AF.

The main strength of this study is the prospective evaluation in two large community-based cohort studies that used comparable methods for ascertaining incident AF, ischemic stroke and CV death. In addition, only incident AF cases were considered, thus, reducing potential survival bias that could have arisen if prevalent cases were included. Other strengths include long-term follow up with meticulous adjudication of the outcome, and the proximity of exposure and covariate information to the time of incident AF. Several limitations need to be considered. First, non-hospitalized stroke events that were not validated in the studies may have influenced the results; however, the magnitude of any potential underestimation of the rate of stroke is likely to be small (<5%). Second, while the classification of smoking used in our study provided a simple characterization of smoking habits, the amount of smoking exposure, such as using pack-years, could have provided a more valid relationship between smoking and the CV outcome. Third, several studies have highlighted the effect modification of alcohol beverage type on the relationship of alcohol intake and CV outcome.[32] This, however, was not examined in our study due to lack of detailed information. Fourth, in determining the at-risk population in our study, asymptomatic and paroxysmal cases of AF may have been missed by our case ascertainment. Previous studies have indicated acceptable validity of AF ascertainment in the ARIC Study and the CHS, and the incidence rates of AF in the ARIC Study and the CHS are similar to those of other community-based studies.[8, 10, 33, 34] Fifth, we were not powered to evaluate the outcome of ischemic stroke alone. However, research has shown that anticoagulation also reduces the risk of death in patients with AF, suggesting that many AF-related deaths may be related to ischemic stroke.[35] Further, higher CHA_2_DS_2_-VASc score is related to higher risk of death.[6] Hence, it is not unreasonable to evaluate a composite endpoint of ischemic stroke and CV death. Sixth, adjusting for variables contained in CHA_2_DS_2_-VASc, which could represent mediators (vs. confounders) on the causal pathway between smoking, alcohol and obesity (exposure factors) and ischemic stroke or cardiovascular death (outcome), may have led to over-adjustment leading to statistical attenuation of the association.

In conclusion, in two community-based cohort studies, we found that smoking was associated with increased risk of the composite outcome of ischemic stroke or CV death in ARIC, which comprised mostly middle-aged to young-old (65–74 years) adults, but not in CHS, which comprised mostly middle-old or oldest-old (≥75 years) adults with AF. Heavy alcohol consumption and obesity were not associated with adverse CV outcomes in persons with AF. Further investigation is needed to search for other modifiable risk factors that are associated with an increased risk of adverse CV outcomes in individuals with AF.
